# Disrupting the Homeostasis of High Mobility Group Protein Promotes the Systemic Movement of *Bamboo mosaic virus*

**DOI:** 10.3389/fpls.2020.597665

**Published:** 2020-12-16

**Authors:** Mazen Alazem, Meng-Hsun He, Chih-Hao Chang, Ning Cheng, Na-Sheng Lin

**Affiliations:** Institute of Plant and Microbial Biology, Academia Sinica, Taipei, Taiwan

**Keywords:** high mobility group proteins, virus movement, *Bamboo mosaic virus*, plant-virus interaction, nucleoprotein

## Abstract

Viruses hijack various organelles and machineries for their replication and movement. Ever more lines of evidence indicate that specific nuclear factors are involved in systemic trafficking of several viruses. However, how such factors regulate viral systemic movement remains unclear. Here, we identify a novel role for *Nicotiana benthamiana* high mobility group nucleoprotein (NbHMG1/2a) in virus movement. Although infection of *N. benthamiana* with Bamboo mosaic virus (BaMV) decreased *NbHMG1/2a* expression levels, nuclear-localized NbHMG1/2a protein was shuttled out of the nucleus into cytoplasm upon BaMV infection. *NbHMG1/2a* knockdown or even overexpression did not affect BaMV accumulation in inoculated leaves, but it did enhance systemic movement of the virus. Interestingly, the positive regulator Rap-GTPase activation protein 1 was highly upregulated upon infection with BaMV, whereas the negative regulator thioredoxin h protein was greatly reduced, no matter if *NbHMG1a/2a* was silenced or overexpressed. Our findings indicate that NbHMG1/2a may have a role in plant defense responses. Once its homeostasis is disrupted, expression of relevant host factors may be perturbed that, in turn, facilitates BaMV systemic movement.

## Introduction

Viruses hijack various cellular machineries to utilize the molecules, subcellular structures, and trafficking systems required for their replication and movement (Huang et al., 2017a; Pitzalis and Heinlein, 2017). For local movement, viruses move from one cell into neighboring cells through plasmodesmata (PD), whereas viruses enter veins and traffic through the vascular system for systemic movement (Lough and Lucas, 2006; Hipper et al., 2013). Depending on the virus group, mobilized viral forms can be either viral nucleoprotein complexes or virions, with both forms usually being assisted by specific virus-encoded non-structural proteins (Hipper et al., 2013; Solovyev and Savenkov, 2014; Pitzalis and Heinlein, 2017). In the Potexvirus group, such proteins are encoded by triple gene block (TGB), which are translated into three movement proteins (MPs) TGBp1, 2, and 3 (Solovyev et al., 2012).

Ever more lines of evidence indicate that the translocation of viral MPs to nuclei, where they interact with specific nucleoproteins, is an essential step in promoting systemic viral infection (Lukhovitskaya et al., 2013; Solovyev and Savenkov, 2014; Lukhovitskaya et al., 2015). These interactions lead to transcriptional programming that reregulate the cell to confer optimal conditions for virus replication and spread (Solovyev and Savenkov, 2014). In addition, a few studies have reported that entry of specific viral proteins into the nucleus results in the export of specific host nuclear proteins to the cytoplasm (Krichevsky et al., 2006; Chang et al., 2016; Li et al., 2018). However, depending on the type of exported protein, this relocalization might be virus-regulated to assist trafficking or be a plant defense reaction that triggers an immune response. We previously identified the nucleolar protein fibrillarin as being a critical factor required for autonomous movement of the satellite RNA of *Bamboo mosaic virus* (satBaMV). The satBaMV-encoded P20 protein forms a complex with fibrillarin in the nucleolus and punctate structures associated with PDs (Chang et al., 2016). However, export of other nuclear proteins beyond the nucleus might represent a plant response to invading pathogens. For example, Arabidopsis high mobility group (HMG) protein B3 is exported to the apoplast where it functions as a damage-associated molecular pattern (DAMP), recognizes avirulent factors of the nectrophic fungus *Botrytis cinerea*, and triggers defenses mediated by salicylic acid (SA) (Choi et al., 2016).

To date, a few nuclear factors have been identified as being involved in the systemic movement of certain viruses (Hipper et al., 2013; Li et al., 2018). A previous study demonstrated that translocation of TGBp1 from *Potato mop-top virus* (PTMV) into the nucleus, mediated by the shuttle protein importin-α, is important for systemic trafficking of the virus, with importin-α knockdown reducing viral accumulation in upper leaves (Lukhovitskaya et al., 2015). However, it is not known how TGBp1 functions in the nucleus to enable PTMV to move systemically. In another example, TGBp1 of *Barley stripe mosaic virus* (BSMV) interacts with and exports fibrillarin-2 (Fib2) to PD. Fib2 also associates with the ribonucleoprotein (RNP) movement complex of BSMV, providing direct evidence of the ability of viral TGBp1 to hijack and employ Fib2 for BSMV cell-to-cell movement (Li et al., 2018). Previous works have indicated that such nuclear factors may regulate transcriptome profiles, inducing other factors required for systemic movement (Solovyev and Savenkov, 2014). Interestingly, several such nuclear factors are nucleolus-related proteins (Hiscox, 2007). For example, interaction of *Groundnut rosette virus* (GRV) open reading frame 3 (ORF3) with fibrillarin in the nucleolus is essential for re-localization of fibrillarin to the cytoplasm, as well as for the assembly of cytoplasmic RNP particles (ORF3-RNA-Fibrillarin) required for long-distance movement (Kim et al., 2007).

HMG proteins represent a heterogeneous class of ubiquitous and relatively abundant non-histone proteins associated with chromatin. They modulate chromatin structure and act as architectural factors in the assembly of nucleoprotein (Wu et al., 2003; Bianchi and Agresti, 2005; Reeves, 2010). HMGAs (previously known as HMG1s) bind specifically to AT-rich DNA through a small DNA binding motif, termed the AT-hook, and they are involved in diverse nuclear and cellular processes, including gene transcription (Lewis et al., 2001; Reeves and Beckerbauer, 2001; Bianchi and Agresti, 2005; Reeves, 2010). A role for HMGs in host-pathogen interactions has been reported previously (Choi et al., 2016; Sprague et al., 2018), playing a positive role in host defense against fungal or viral pathogens. For example, *Arabidopsis thaliana* HMGB3 (AtHMGB3) was identified as a DAMP molecule that mediates innate immune responses upon infection with *B. cinerea* (Choi et al., 2016). AtHMGB3 translocalizes to the apoplast upon infection with *B. cinerea*, activates MAPK, induces callose accumulation, and triggers the expression of defense-related genes, which collectively reduces levels of *B. cinerea*. Silencing AtHMGB3 increased *A. thaliana* susceptibility to *B. cinerea* (Choi et al., 2016). Similarly, the mouse cell lines NIH-3T3 and 3T6-Swiss secreted HMGB1 upon infection with *Herpes simplex virus* strain HSV1716, an oncolytic herpes virus. An HMGB1 knockdown cell line revealed its role in restricting HSV1716, indicating that it acts as a DAMP to regulate pro-inflammatory cytokine release and inflammation (Sprague et al., 2018; Hong et al., 2019).

Focusing on host nuclear factors is an emerging field of study in host-pathogen interactions, yet their involvement in viral movement remains largely unexplored (Solovyev and Savenkov, 2014). Here, we used BaMV to investigate the role of *Nicotiana benthamiana* HMGs (NbHMGs) in systemic movement of BaMV. BaMV is a flexuous Potexvirus with a single-stranded positive-sense RNA genome of 6.4 kb that encodes five ORFs (Lin et al., 1992; DiMaio et al., 2015; Hsu et al., 2018). The first ORF encodes a 155 kDa protein that functions as a replicase with methyltransferase (Li et al., 2001a; Huang et al., 2004), helicase (Li et al., 2001b) and RNA-dependent RNA polymerase (RdRp) domains (Li et al., 1998). The second to fourth ORFs encode three TGBp proteins; TGBp1 of 28 kDa, TGBp2 of 13 kDa, and TGPp3 of 6 kDa, respectively (Lin et al., 1994; Yang et al., 1997). These TGBp proteins assist in cell-to-cell movement of BaMV (Lin et al., 2006; Hsu et al., 2008; Chou et al., 2013). The fifth ORF encodes a 25 kDa capsid protein (CP) that is involved in virus encapsidation, symptom formation (Lan et al., 2010), and viral movement (Lee et al., 2011). When BaMV replicates, three major RNAs are generated; a genomic RNA (gRNA) of 6.4 kb and two sub-genomic RNAs (sgRNA) denoted sgRNA1 and sgRNA2 of 2 and 1 kb, respectively (Lin et al., 1994; Yang et al., 1997).

In this study, we identified two *HMG1* genes in *N. benthamiana* in which only *NbHMG1/2a* was detectable and downregulated upon infection with BaMV. NbHMG1/2a localized exclusively in the nucleus but, upon BaMV infection, it was also detected in the cytoplasm. Knockdown or overexpression of *NbHMG1/2a* did not affect BaMV accumulation in inoculated leaves. Interestingly, under both scenarios (*NbHMG1/2a*-silenced or -overexpressed), we observed significantly enhanced systemic BaMV. Moreover, previously identified host factors required for BaMV cell-to-cell and systemic movement were not affected by the status of HMG in BaMV-infected plants, except for two important host factors–Rap-GTPase activation protein 1 (Rap-GAP1) (Huang et al., 2013) and thioredoxin h protein (TRXh2) (Chen et al., 2018)–that played a positive and negative role, respectively, in local and systemic movement of BaMV. These results provide evidence for the role of HMG protein in systemic movement of an RNA virus.

## Results

### Orthologs of *Arabidopsis* HMGB3 in *N. benthamiana*

**FIGURE 1:**
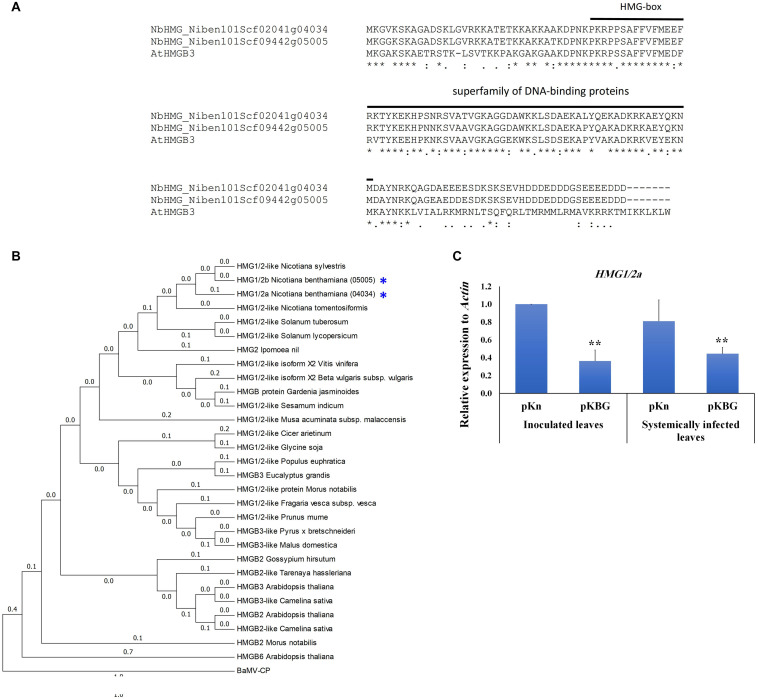
Phylogenetic and expression analyses of *Nicotiana benthamiana HMG1/2* genes. **(A)** Similarities of *NbHMG* accessions to *Arabidopsis thaliana HMGB3*. Two *NbHMG* accessions with high similarity to *AtHMGB3* were obtained by blasting the AtHMGB3 coding sequence against the *N. benthamiana* database (Sol Genomics Networks), i.e., NbHMG_101Scf02041g04034 (*NbHMG1/2a*) and NbHMG_101Scf09442g05005 (*NbHMG1/2b*). **(B)** Phylogenetic analysis of *NbHMG1/2* with orthologs from other species. The HMG phylogeny was generated using the Neighbor Joining method in MEGA 6.0 software. Numbers represent relative phylogenetic distance. BaMV capsid protein (CP) was used as an outgroup to root the tree. **(C)** Relative expression levels of *NbHMG1/2a* by RTqPCR in *N. benthamiana* plants infected with BaMV infectious clone (pKBG) compared with plants infiltrated with empty vector (pKn). Inoculated leaves were tested at 3 days post infiltration (dpi), and the systemic leaves were tested at 6 dpi. Actin was used as an internal control. Data represent the mean ± standard deviation from three biological replicates. One-sided student *t-*tests were performed to determine significant differences. Asterisks indicate significant differences relative to control lines (infiltrated with pKn), with ** representing *P* < 0.01.

In order to identify the orthologs of *AtHMGB3* in *N. benthamiana*, we blasted the coding sequence of *AtHMGB3* against *N. benthamiana* genome database v1.0.1 in the Sol Genome network (solgenomics.net). The blast analysis identified two accessions; Niben101Scf09442g05005.1 and Niben101Scf02041g04034.1 with 86.55 and 82.35% identities covering 103 and 98 amino acids (aa), respectively, of the 119-aa *AtHMGB3* sequence (Figure 1A). Both sequences show high identity to each other in the coding region (CDS) (95%) (Figure 1A) and in the 5′ untranslated region (UTR) (93%), but share low similarity in the 3′ UTR (43.5%) (Supplementary Figure S1). The region of high similarity corresponds to the high mobility group box domain (Figure 1A). However, our phylogenetic analysis of HMG orthologs from different species clustered these two accessions in close proximity to *HMG1/2* from different *Nicotiana* and *Solanum* species, prompting us to denote the two accessions Niben101Scf02041g04034.1 and Niben101Scf09442g05005.1 as *NbHMG1/2a* and *b*, respectively (Figure 1B). We then measured the expression levels of *NbHMG1/2a* and *b* in *N. benthamiana* plants in response to BaMV infection. Although expression of *NbHMG1/2a* was significantly decreased upon BaMV infection in both inoculated and systemic leaves at 6 days post infection (dpi) (Figure 1C), we did not detect any expression of *NbHMG1/2b* using different pairs of primers targeting the CDS and 3′ UTR under various conditions [healthy plants or plants infected with BaMV infectious clone pKBG or infiltrated with empty vector (EV) of the *Tobacco rattle virus* (TRV) (TRV-EV)]. These data suggest that *NbHMG1/2a* might be involved in the interaction between BaMV and *N. benthamiana*, but *NbHMG1/2b* may not be expressed in *N. benthamiana* leaves under normal growth conditions or when it is under viral attack.

### NbHMG1/2a Localizes in the Nucleus and Nucleolus

**FIGURE 2:**
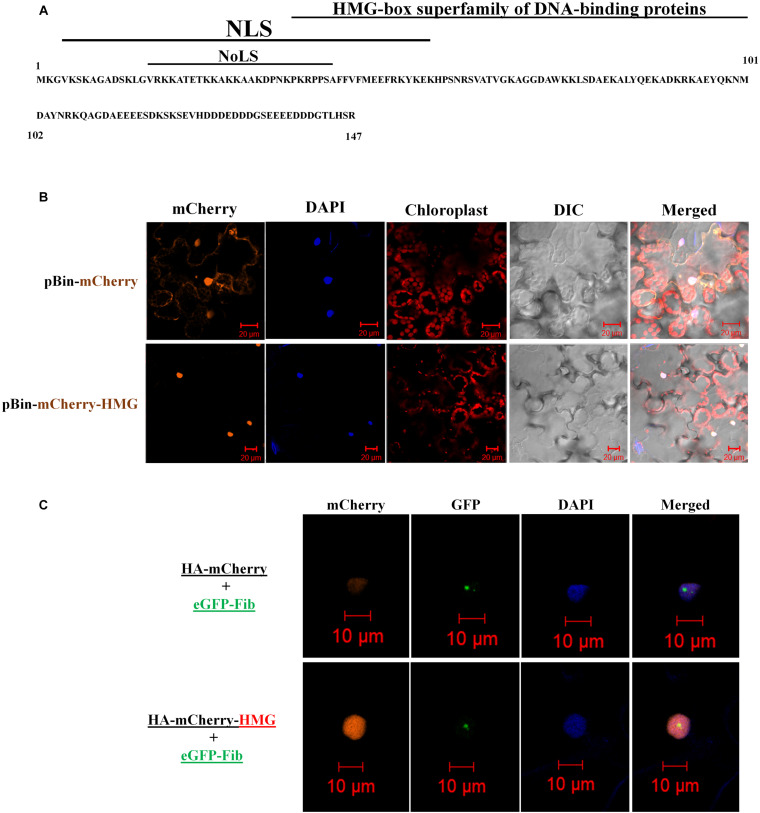
Domain analysis and localization of NbHMG1/2a. **(A)** Predicted domains encoded in NbHMG1/2a: a nuclear localization signal (NLS) between 4 and 56 amino acids (aa), a nucleolar localization signal (NoLS) between 15 and 42 aa, and a DNA-binding domain between 36 and 101 aa. No nuclear export signal was found. **(B)** NbHMG1/2a localizes in the nucleus. *N. benthamina* leaves were infiltrated with *Agrobacterium* carrying pBin61-HA-HMG-mCherry or with control vector pBin61-HA-mCherry. **(C)** NbHMG1/2a localizes in the nucleolus. *N. benthamina* leaves were infiltrated with *Agrobacterium* carrying HA-mCherry and nucleolus-localized fibrillarin fused with eGFP (eGFP-Fib), or with HA-mCherry-HMG and eGFP-Fib. DAPI was used for nuclear staining. Images were taken 3 days after infiltration, and bars represent a measurement scale of 20 and 10 μm for the nucleus and nucleolus, respectively. The experiment was repeated three times with similar results and representative images are shown.

Next, we identified the functional domains of NbHMG1/2a. The conserved domain database of NCBI (Marchler-Bauer et al., 2015) predicted a DNA binding domain in a motif corresponding to aa 36-101 of NbHMG1/2a protein, with an *E*-value of 8.37e-15 (Figure 2A). A nucleus localization signal (NLS) and a nucleolus localization signal (NoLS) were also predicted in motifs at 4–56 and 15–42 aa, respectively, using cNLS and NoD mappers (Kosugi et al., 2009b; Scott et al., 2011; Figure 2A). No nucleus export signal was identified in the coded protein using the LocNES server (Xu et al., 2015), but the NetNES 1.1 server (la Cour et al., 2004) predicted a weak signal (below threshold) in a motif at 44–50 aa. To confirm these findings, we cloned the full *NbHMG1/2a* coding sequence with a 3HA-mCherry tag into the pBin61 expression vector (Chang et al., 2016). Transient expression of NbHMG1/2a in *N. benthamiana* leaves showed that this protein clearly localizes in the nucleus (Figure 2B), the nucleolus, and the nucleoplasm (Figure 2C), but no cytoplasmic localization was detected across 3 days of observation (1.5, 2, and 3 dpi) (Supplementary Figures S2–S4). Thus, under normal plant growth conditions, NbHMG1/2a localizes in the nucleus and nucleolus.

### Both Silencing and Overexpression of *NbHMG1/2a* Increase Systemic Movement of BaMV

**FIGURE 3:**
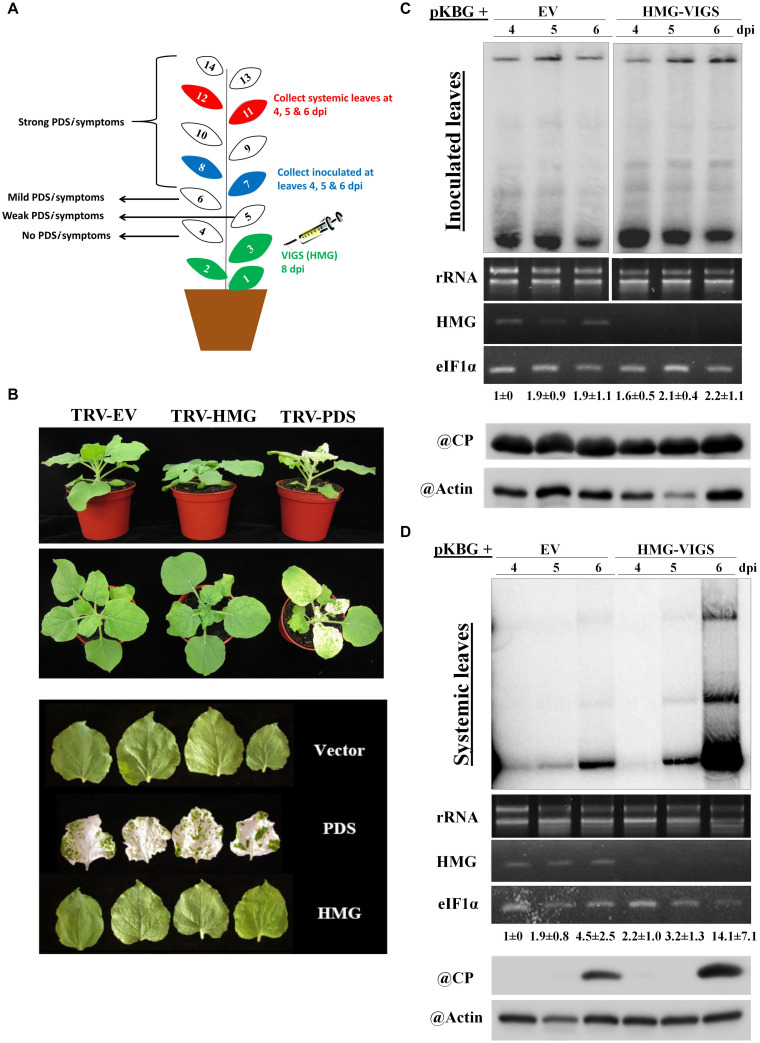
Effects of silencing *NbHMG1/2a* on plant growth and resistance to BaMV. **(A)** Schematic illustration of our VIGS experimental design. The first three leaves of *N. benthamiana* plants were infiltrated with the silencing vector TRV carrying the *NbHMG1/2a* fragment or control mCherry fragment. Eight days later, leaves 7 and 8 were infiltrated with pBin61 carrying BaMV (pKBG). Inoculated leaves (leaves 7 and 8) and systemic leaves (leaves 11 and 12) were collected at 3, 4, and 6 days after BaMV infection (dpi). **(B)** Phenotype of *NbHMG1/2a*-silenced plants. Plants infiltrated with TRV-mCherry, TRV-HMG, and TRV-PDS. Photos were taken 8 days after agroninfiltration with TRVs. Upper and middle panel show plant height and spread, and lower panel shows leaf size. **(C,D)** RNA blot of BaMV in the inoculated leaves **(C)** and systemic leaves **(D)** of control plants (infiltrated with TRV-mCherry) and *NbHMG1/2a*-silenced plants (HMG-VIGS). The second panel represents rRNA levels as internal control. The third panel shows *NbHMG1/2a* levels in the control and silenced plants. eIF1α was used as an internal control. Values represent average accumulation of BaMV genomic RNA from three biological replicates ± standard deviation. Lower panels represent protein blots of BaMV capsid protein (CP) (25 kDa) and actin in the control and silenced plants.

We examined the effects of silencing *NbHMG1/2a* to determine if it has any effect on BaMV accumulation or spread. To avoid silencing any off-target genes, we employed the virus-induced gene silencing (VIGS) tool in the Sol genomics database to assess the full CDS of *NbHMG1/2a* (Fernandez-Pozo et al., 2015). The VIGS tool recommended using any fragment within the first 300 base pairs (bp) of the *NbHMG1/2a* CDS to avoid silencing off-target genes (Supplementary Table S2). A 21-mer siRNA generated from this silencing construct would have large numbers of matches with both *NbHMG1/2a* (404 matches) and *NbHMG1/2b* (101 matches), but no other potential genes were identified as being off-target (except for two accessions each with one match) (Supplementary Table S2). To silence *NbHMG1/2a*, TRV silencing vector carrying *NbHMG1/2a* partial fragments was agroinfiltrated into 18-day-old *N. benthamiana* plants to silence *NbHMG1/2a.* The *Phytoene desaturase* (*PDS*) gene acted as an indicator of silencing efficiency and mCherry functioned as an EV negative control. After a strong bleaching phenotype appeared on *PDS*-silenced plants, the upper leaves (leaves 7 and 8) were agroinfiltrated with pKBG (Figure 3A). Silencing of *NbHMG1/2a* resulted in a stunted phenotype compared to EV- or *PDS*-silenced plants (Figure 3B). Nonetheless, leaf size was similar among control and silenced plants (Figure 3B). Levels of BaMV RNA or CP were comparable in inoculated leaves at 4, 5, and 6 dpi among EV- and *NbHMG1/2a*-silenced plants (Figure 3C). However, BaMV spread more rapidly to the upper systemic leaves (leaves 11 and 12) and exhibited greater accumulation (∼3.1-fold relative to EV-silenced plants) in *NbHMG1/2a*-silenced plants at 6 dpi (Figure 3D). To confirm these results, we carried out RTqPCR for samples collected at 6 dpi (Supplementary Figure S5A). Using primers targeting BaMV genomic RNA (RNA-dependent RNA polymerase “RDR”), the RTqPCR showed that *RDR* accumulated equally in the BaMV-inoculated leaves of EV and the *NbHMG1/2a*-silenced plants, but showed ∼ 6.7-fold upregulation of *RDR* in the systemically infected leaves (Supplementary Figure S5A). We next tested whether the TRV silencing vector is affected by BaMV or the reduced level of *NbHMG1/2a*. While TRV-RDR accumulated equally in the control and BaMV inoculated leaves, TRV-RDR exhibited 50% increase in the systemically infected leaves (Supplementary Figure S5B). Of note, VIGS resulted in significant decrease of NbHMG1/2a in both BaMV-inoculated leaves as well as in the systemically infected leaves (Supplementary Figure S5C). This indicates that BaMV infection may allow faster spread of the silencing vector TRV.

**FIGURE 4:**
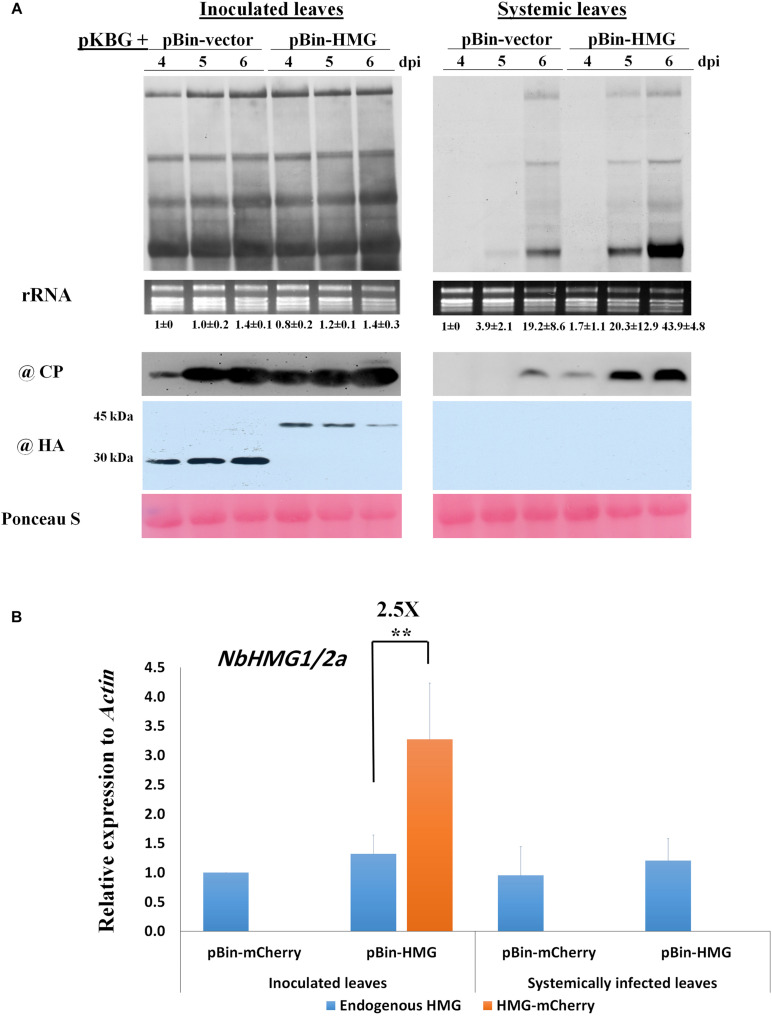
Overexpression of *NbHMG1/2a* enhances systemic movement of BaMV. **(A)** Twenty-four-day-old *N. benthamiana* plants were co-agroinfiltrated with pKBG and pBin-HA-mCherry or pBin-HA-mCherry-HMG. Samples were collected from inoculated and systemic leaves at 4, 5, and 6 dpi. Upper panels are Northern blots for BaMV, with rRNA used as a loading control. Lower panels are protein blots of BaMV CP and HA-tag to detect HA-mCherry (30 kDa) and HA-mCherry-HMG (45 kDa). Ponceau S was used as a loading control. The experiment was conducted with three biological replicates, which generated similar results. Values below Northern blots represent average accumulation of BaMV genomic RNA from three biological replicates ± SD. **(B)** Relative expression of endogenous *NbHMG1/2a* and the overexpressed gene *NbHMG1/2a-mCherry* in inoculated and systemic leaves of *NbHMG1/2a*-overexpressing plants based on RTqPCR. According to one-sided student *t-*test, asterisk indicates significant difference for the expression of *NbHMG1/2a-mCherry* relative to the endogenous *NbHMG1/2a*, with ** representing *P* < 0.01. Actin was used as an internal control. Error bars represent the standard deviation from three biological replicates.

Since *NbHMG1/2a*-silenced plants displayed enhanced systemic movement and accumulation of BaMV, we assessed the impact of transient overexpression of *NbHMG1/2a* (fused with HA-mCherry tag) on both local and systemic BaMV accumulation. Although levels of BaMV RNA and CP protein were similar in the inoculated leaves of control and overexpressing plants, accumulation of BaMV was enhanced (∼3.6-fold relative to control) in the systemically infected leaves at 6 dpi (Figure 4A). To confirm these results, BaMV-*RDR* expression level was measured at 6 dpi using RTqPCR (Supplementary Figure S6). BaMV-RDR showed no difference in the inoculated leaves of the control and NbHMG1/2a-overexpressing plants. However, the accumulation level was threefold upregulated in the systemically infected leaves overexpressing NbHMG1/2a compared with the infected control (Supplementary Figure S6). Notably, the overexpressed *NbHMG1/2a* was detectable in inoculated leaves (Figure 4A), and it did not result in systemic silencing of *NbHMG1/2a* as RT-qPCR revealed that transcript levels of the endogenous *NbHMG1/2a* were comparable between control and overexpressing plants, and the overexpressed gene accumulated 2.5-fold more than the endogenous gene in the inoculated leaves (Figure 4B). These results confirmed that transcript level of *NbHMG1/2a* was boosted by transient expression in the inoculated leaves.

### Host Factors Required for BaMV Systemic Movement Are Regulated in *NbHMG1/2a*-Silenced and -Overexpressing Plants

**FIGURE 5:**
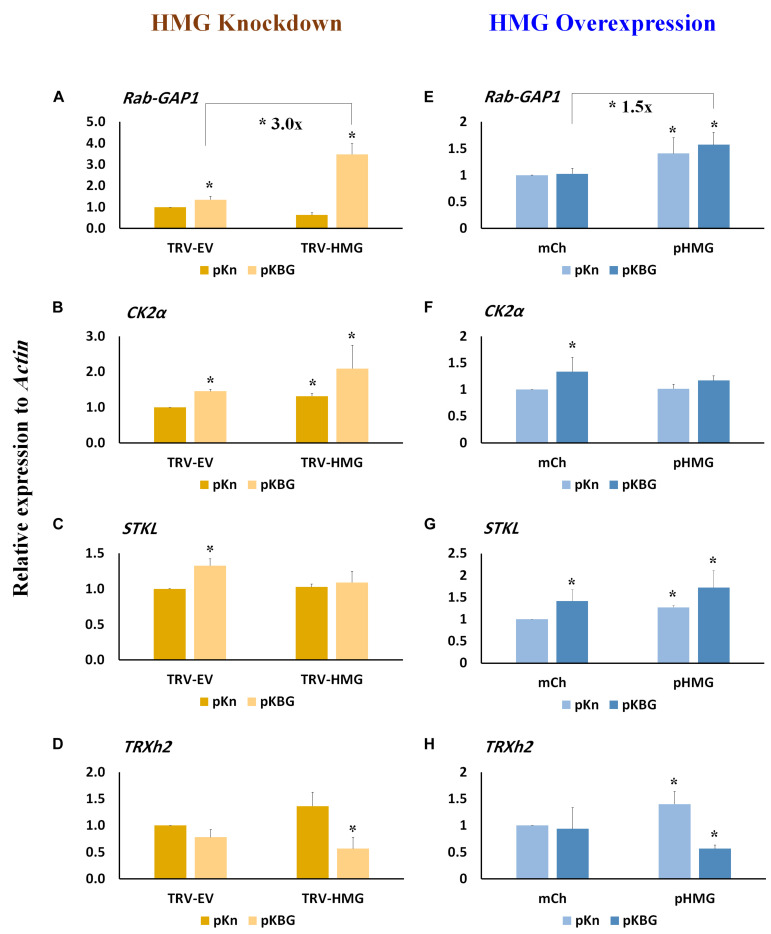
Relative expression of host genes involved in BaMV movement in *NbHMG1/2a-* knockdown and -overexpressing plants. Transcript levels of *RAB-GAP1* **(A,E)**, *CK2*α **(B,F)**, *STKL* **(C,G)**, and *TRXh2* **(D,H)** in *N. benthamiana* plants infected with pKBG or pKn and upon *HMG* being knocked-down or overexpressed, respectively. Expression level was measured in the BaMV-inoculated leaves at 6 dpi in both HMG-silenced and HMG-overexpressed plants. Actin was used as an internal control. Data represent the mean ± SD from three biological replicates. One-sided Student’s *t-*tests were performed to determine significant differences. Asterisks indicate significant differences relative to mock lines (infiltrated with pKn and pBin-mCherry), with * representing *P* < 0.05.

Several host factors required for BaMV cell-to-cell and systemic movement have been identified previously (Cheng, 2017; Huang et al., 2017a). These factors include the activating protein Rab GTPase (*Rab-GAP1*) (Huang et al., 2013), casein kinase 2α (*CK2*α) (Hung et al., 2014), serine/threonine kinase-like protein (*STKL*) (Cheng et al., 2013), and thioredoxin h protein (*TRXh2*) (Chen et al., 2018). *TRXh2* plays a negative role in BaMV accumulation, whereas the other genes enhance local BaMV accumulation, with *RAB-GAP1* increasing BaMV accumulation in both inoculated and systemic leaves (Huang et al., 2013). In BaMV-inoculated leaves, we found that expression of *Rab-GAP1, CK2*α, and *STKL* all increased following BaMV infection of TRV-EV control plants (Figures 5A–C), whereas expression of *TRXh2* remained unchanged (Figure 5D). In *NbHMG1/2a-*silenced plants (*TRV-HMG*), only *CK2*α presented a slight increase in expression compared to TRV-EV control (Figure 5B). However, upon BaMV infection of *NbHMG1/2a-*silenced plants, *Rab-GAP1* was the only gene exhibiting a significant increase in expression relative to TRV-EV control plants (Figure 5A). Of note, expression of *TRXh2* decreased in TRV-HMG-agroinfiltrated plants compared to TRV-EV control plants (Figure 5D).

Expression of *Rab-GAP1*, *STKL*, and *TRXh2* was significantly increased in plants transiently overexpressing *NbHMG1/2a* (pHMG) compared to plants infiltrated with the mCherry-expressing vector (mCh) (Figures 5E,G,H), whereas that of *CK2*α was not affected (Figure 5F). After BaMV infection, *Rab-GAP1* and *STKL* were upregulated (Figures 5E,G), and *TRXh2* was downregulated (Figure 5F). Only *Rab-GAP1* was significantly increased in BaMV-infected pHMG-overexpressing plants compared to mCh-infected ones (Figure 5E). These findings indicate that only *Rab-GAP1* was significantly increased in both silenced and overexpressing plants following BaMV infection. Moreover, *TRXh2* was downregulated (∼ 2.5-fold) in the infected pHMG-overexpressing plants when compared to mCh-infected plants (Figure 5H). Together, these findings imply that enhanced systemic accumulation of BaMV may be attributable to coordinated upregulation of *Rab-GAP1* expression and downregulation of *TRXh2*. Previously, it was proposed that Rab-GAP1 triggers one of the RabGTPases to release vesicles containing the BaMV-movement complex for trafficking to the PD (Huang et al., 2013, 2017a). The speed at which viral movement complex is delivered to the PD affects the onset and swiftness of systemic trafficking (Rodrigo et al., 2014).

### BaMV Infection Triggers Relocalization of NbHMG1/2a to the Cytoplasm

**FIGURE 6:**
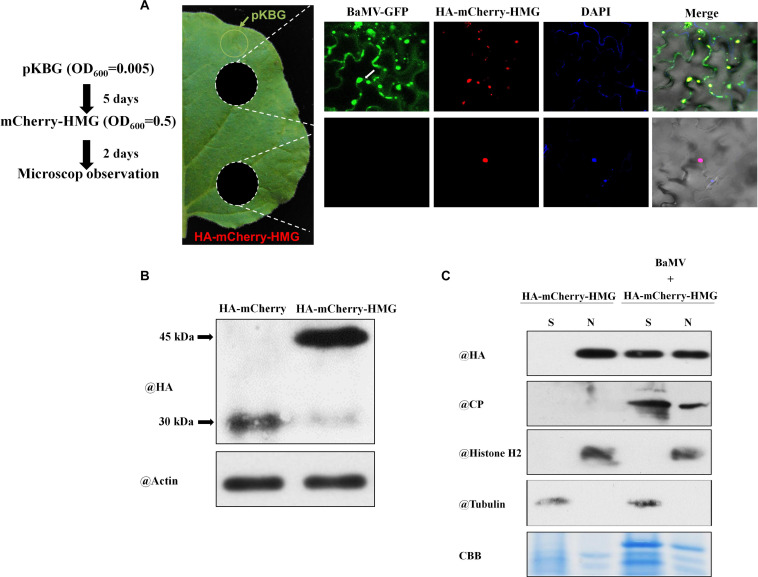
Localization of NbHMG1/2a in BaMV-GFP-infected *N. benthamiana*. **(A)** Leaves of *N. benthamiana* were agroinfiltrated with pKBG (BaMV-GFP) (OD = 0.005) into the region represented by the green circle. Five days later, inoculated leaves were agroinfiltrated with mCherry-HMG (OD = 0.5) in whole leaves. Samples were excised (white circles) 2 days afterward for confocal microscopic observation. To detect the nucleus, 4′,6-diamidino-2-phenylindole (DAPI) solution was infiltrated into the *N. benthamiana* leaves before observation. Scale bars are 50 μm. Arrows and arrowheads indicate nucleus and cytoplasm, respectively. The experiment was repeated three times with similar results, and representative images are shown. **(B)** Protein gel blot analysis of HA-mCherry and HA-mCherry-HMG at 2 dpi by anti-HA or anti-actin body. **(C)** Protein gel blot analysis of HA-mCherry-HMG in subcellular fractions of BaMV infected leaf tissues. Histone H2 was used as a nuclear marker, and cytosolic tubulin served as a cytosolic marker. N, nuclear protein extracts; S, total protein extracts depleted of nuclei.

We have shown that NbHMG1/2a localizes exclusively in the nucleus (Figure 2). This observation was confirmed at 1.5, 2, and 3 dpi (Supplementary Figures S4–S6), and BaMV did not appear to affect this localization within these timeframes. We tried to extend our observations to the late stage of infection, but levels of transiently-expressed proteins had greatly declined, resulting in undetectable signal at 6 dpi (Supplementary Figure S7). To overcome this problem, we agroinfiltrated very limited amounts of pKBG (BaMV infectious clone carrying GFP) (OD_600_ = 0.005) into a defined region of leaves (Figure 6A, green circle). mCherry-HMG (OD_600_ = 0.5) was then expressed in the whole leaves. Two days later, GFP fluorescent signals were only detected in close proximity to the pKBG-infiltrated regions (upper white circle), but no fluorescent signals were observed in the distal cells (lower white circle) (Figure 6A). Surprisingly, in cells showing BaMV-GFP infection, mCherry-HMG signals were not only detected in the nucleus but also in cytosol (Figure 6A, upper panel). However, mCherry-HMG signals remained localized in the nucleus where no BaMV-GFP signals were observed (Figure 6A). To confirm the mCherry-HMG is localized in both the cytosol and the nucleus following BaMV-GFP infection, cell fractionation was carried out to separate the nuclei from the cytoplasm. First, *N. benthamiana* leaves were infiltrated with HA-mCherry or HA-mCherry-HMG, and the HA signal in the infiltrated tissues was detected by protein blot at 48 h after infiltration (Figure 6B). We next purified nuclei from control and the BaMV-infected leaves at 7 dpi, and the presence of HMG, CP, Histone H2 (a nuclear marker protein), and Tubulin (a cytoplasmic marker protein) was determined by protein blots from nuclear extracts. HMG and CP were found in the nuclear and the supernatant fractions, whereas Histone H2 was detectable only in the nuclear fraction, and Tubulin was found only in the supernatant fraction (Figure 6C). We conclude that NbHMG1/2a is translocalized to the cytoplasm upon BaMV infection. Using a similar approach, we observed that a substantial amount of mCherry-HMG moved from the nucleus to cytoplasm upon infection with pKBG (Supplementary Figure S8), whereas mCherry-HMG remained in the nucleus upon pKn infiltration (Supplementary Figure S8), indicating that the relocalization of mCherry-HMG is triggered by BaMV infection.

### No Evidence of Interaction Between Plant NbHMG1/2a and Viral CP or TGBp1 Proteins

Previously, TGBp1 and CP proteins were reported to localize in the nucleus and cytosol and that they play crucial roles in BaMV cell-to-cell and systemic movement (Lin and Chen, 1991; Chang et al., 1997; Palani et al., 2009). Our observations of the localization of NbHMG1/2a in the nucleus and its trans-localization to the cytoplasm prompted us to test if CP or TGBp1 interacts with NbHMG1/2a. However, yeast two-hybrid analysis revealed that neither of these BaMV proteins directly interacts with NbHMG1/2a (Supplementary Figure S9), suggesting that the effect of BaMV on NbHMG1/2a localization is indirect. These observations imply that NbHMG1/2a may not be involved in regulating cell-to-cell movement of BaMV, but it might have other functions related to anti-BaMV defenses.

## Discussion

Entry of specific viral proteins into the nucleus and their interaction with certain nuclear or nucleolar proteins are critical processes for the systemic movement of several viruses (Solovyev and Savenkov, 2014). Yet, how these interactions locally or systemically alter plant mechanisms has not been fully uncovered, despite a few studies suggesting transcriptional reprogramming conditions the cell for enhanced viral replication and movement (Chen et al., 2013; Levy et al., 2013; Solovyev and Savenkov, 2014).

Cell-regulated export of certain nuclear proteins in response to viral infection is intriguing and implies specific functions after viral attack. Taking HMG proteins as an example, several works have reported roles for these proteins in chromatin modification and transcriptional regulation, requiring nuclear localization (Ju et al., 2006; Launholt et al., 2006; Antosch et al., 2012). However, upon infection with *B. cinerea*, export of AtHMGB3 to the apoplast has been deemed critical for initiation of innate immunity. AtHMGB3 acts as a DAMP molecule that recognizes avirulent factors of *B. cinerea* to trigger SA-mediated defenses (Choi et al., 2016). Here, our data also shows that NbHMG1/2a is normally localized in the nucleus (Figure 2), but substantial amounts of NbHMG1/2a can be detected in the cytoplasm but not at PDs in response to BaMV late infection (Figure 6), potentially excluding a direct role of NbHMG1/2a in BaMV cell-to-cell movement. Our observation that *NbHMG1/2a* expression is reduced upon BaMV infection indicates that the virus may exert this effect to modulate the host for optimal infection conditions (Figure 1C). Indeed, this possibility is supported by our finding that systemic spread of BaMV was faster in *NbHMG1/2a*-silenced plants relative to control plants (Figure 3). In addition, neither CP nor TGBp1, both of which localize in the nucleus and cytoplasm, were found to interact directly with NbHMG1/2a (Supplementary Figure S9). These findings do not exclude the notion that NbHMG1/2a relocalization to the cytosol is defense-related. In fact, other HMGs, such as mouse HMGB1, contribute to cell defense against the HSV1716 oncolytic herpes virus (Sprague et al., 2018). Overall, studies of HMGs from different species suggest several members of this protein group function in defense against pathogens.

Expression levels of host factors required for local and systemic movement of BaMV indicate that Rab-GAP1 might be regulated by NbHMG1/2a because levels of this protein increased when NbHMG1/2a was transiently overexpressed (Figure 5E), but remained unaffected in *NbHMG1/2a*-silenced plants (Figure 5A). However, in response to BaMV infection, expression levels of *Rab-GAP1* were upregulated 3-fold in *NbHMG1/2a*-silenced plants (Figure 5A). It was reported previously that silencing of *Rab-GAP1* drastically reduced systemic and local BaMV accumulation by ∼98 and 50%, respectively, but had no effect on protoplast levels (Huang et al., 2013). Thus, Rab-GAP1 serves as a positive regulator of BaMV movement. In contrast, BaMV infection reduced the expression of *TRXh2* only in *NbHMG1/2a*-silenced plants but not in EV-control plants. NbTRXh2 may target TGBp2, disrupting its structural integrity and its association with TGBp1 and TGBp3 (Chen et al., 2018). Regulation of these factors may explain the enhanced systemic accumulation of BaMV in *NbHMG1/2a*-silenced plants and it suggests a role for NbHMG1/2a in transcriptional regulation of Rab-GAP1 and TRXh2, and probably for other factors necessary for the BaMV infection cycle. Previous works have reported roles for HMGs in transcription (Reeves and Beckerbauer, 2001; Ju et al., 2006; Launholt et al., 2006; Reeves, 2010; Antosch et al., 2012), strengthening the notion that other host factors involved in BaMV systemic movement might also be transcriptionally regulated by HMG.

Interestingly, transient overexpression of *NbHMG1/2a* also enhanced systemic movement of BaMV, but it did not affect its local accumulation (Figure 4). Levels of the positive regulator of BaMV movement, Rap-GAP1, were significantly increased upon BaMV infection (Figure 5E), whereas *TRXh2* that impedes systemic BaMV movement was downregulated in infected plants overexpressing *NbHMG1/2a* (Figure 5H). Thus, coordinated upregulation of *Rab-GAP1* expression and downregulation of *TRXh2* may explain the enhanced systemic accumulation of BaMV. It could be speculated that BaMV affects the transcription-related function of NbHMG1/2a because BaMV enhanced the expression of *Rab-Gap1* in the overexpression and silenced plants (Figures 5A,E). While BaMV reduces the expression level of *NbHMG1/2a* (enhanced by the VIGS-mediated transient silencing), BaMV forces the remaining *NbHMG1/2a* to translocalize from the nucleus to the cytosol (Figure 6). Both cases resulted in increased expression of Rab-GAP1, which may suggest that NbHMG1/2a may act as a negative regular for *Rap-GAP1* in the presence of BaMV. The VIGS results support this notion, however, further experimental validations are required to explain how NbHMG1/2a affect the expression of several host factors with/without BaMV infection.

While it could be interpreted that a homeostatic imbalance of HMG affects the expression of factors involved in BaMV movement, these findings may also rule out the potential involvement of antiviral defenses (e.g., callose deposition, antiviral RNA silencing pathway, accumulation of reactive oxygen species, and salicylic and abscisic acids) on systemic BaMV movement. The impacts of these antiviral defenses would be apparent for both inoculated and systemic leaves, as well as at cellular levels (Alazem et al., 2017, 2019; Huang et al., 2019). However, since the effect of NbHMG1/2a was only significant for systemic leaves, those antiviral defenses might not be affected by HMG homeostasis. In a similar example where silencing and overexpression of specific genes induced plant susceptibility to viral infection, a previous study showed that the chloroplast gene *Increased Size Exclusion Limit 2* (*ISE2*) enhanced systemic accumulation of *Tobacco mosaic virus* (TMV) and *Turnip mosaic virus* (TuMV) under conditions of both silencing and overexpression, albeit most likely through different mechanisms (Ganusova et al., 2017). Whereas silencing of *ISE2* enhanced symplastic flux, thereby increasing systemic movement of both TMV and TuMV, levels of the Dicer-like protein of the antiviral RNA pathway was reduced in *ISE2*-overexpressing plants, perhaps explaining the enhanced susceptibility to TMV and TuMV via abrogation of antiviral defense mechanisms (Ganusova et al., 2017). *ISE2* is involved in the chloroplast-to-nucleus retrograde signaling that regulates formation of PD, consequently affecting intercellular viral movement (Ganusova et al., 2020). In our study, the nuclear-localized protein NbHMG1/2a regulates expression of host factors involved in systemic movement of BaMV with no effect observed in the inoculated leaves. The cytoplasmic role of NbHMG1/2a under conditions of BaMV infection warrants further investigation.

## Concluding Remarks

Local and systemic viral trafficking requires specific cellular host factors. These factors vary depending on each stage of movement, such as passage through PDs, movement into companion cells, phloem entry, and exit from the vascular system into the upper leaves. Our study presents evidence of the involvement of a nuclear protein, HMG1/2a, in the systemic movement of plant viruses. HMG1/2a homeostasis affects levels of Rap-GAP1 and TRXh2 to regulate systemic BaMV movement and we speculate that HMG1/2a may affect other factors involved in the infection cycle of BaMV, potentially linked to its reported nuclear function in transcriptional regulation or to its unknown function in the cytoplasm. Viral interactions with host nuclear proteins and subsequent export of these latter into the cytoplasm seem to be diverse phenomena, which are probably infection stage-related. Further studies on how host nuclear factors mediate virus-host interactions will shed light on how viruses control their movement and replication by hijacking host nuclear factors and their downstream pathways.

## Materials and Methods

### HMG Domain Prediction

Nucleus localization signal was predicted using cNLS Mapper with a cut-off score of 5.6^1^ (Kosugi et al., 2009a). Nucleolus localization signal was predicted using NoD with a cut-off score of 0.81/1.00^2^ (Scott et al., 2011). The conserved domains of HMG were defined by querying against conserved domains in NCBI^3^ (Jones et al., 2014; Marchler-Bauer et al., 2015; Marchler-Bauer et al., 2017). The NetNES 1.1 server was used to predict a nuclear export signal, which indicated that a motif at 44–50 aa is responsible for nuclear export, albeit with a weak signal (below threshold) (la Cour et al., 2004).

### Phylogenetic Analysis

Protein sequences of NbHMG1/2 were blasted against the NCBI database. Similar sequences (with at least 70% similarity) were used to generate phylogenetic trees in MEGA 6.0 software and by applying the Neighbor Joining method.

### Plant Growth and Virus Induced Gene Silencing (VIGS)

*N. benthamiana* plants were grown at 28°C in a walk-in plant growth chamber under a 16-h-light/8-h-dark cycle with a white light (Philips TLD 36W/840 ns) intensity of 185–222 μmol m^–2^ s^–1^ at the leaf surface, and a relative humidity of approximately 70%. VIGS was carried out as described previously (Senthil-Kumar and Mysore, 2014). In brief, 18-day-old *N. benthamiana* plants where infiltrated with *Agrobacterium* strain C58C1 carrying either TRV1 or TRV2 vector, with the latter carrying ∼300 bp fragments of NbHMG1 or PDS as a phenotype control. In addition, the TRV2 vector (with mCherry insert) was agroinfiltrated alongside TRV1 as a vector control (TRV-EV). Seven-to-eight days post-infiltration (dpi), the control plants silenced with PDS presented a bleached phenotype, indicating that silencing was effective. Leaves 7 and 8 of *NbHMG1/2a*-silenced plants were later infiltrated with pKBG to assess the effect of *NbHMG1/2a*-silencing on BaMV movement (Liou et al., 2014). pKBG-infiltrated leaves (leaves 7 and 8) and systemically infected leaves (leaves 11 and 12) were collected at 4, 5, and 6 dpi for further analyses. The VIGS experiment was tested in three biological replicates, each of which consisted of three plants.

### Overexpression of *NbHMG1/2a*

Full-length *NbHMG1/2a* was cloned into the expression vector pBin61, with 3xHA-mCherry tagged on the N-terminus of NbHMG1/2a (pBin-HMG). The vector pBin61-3HA-mCherry (pBin-mCherry) was used as a control (Huang et al., 2017b). The BaMV infectious clone pKBG (BaMV construct expressing GFP) (Liou et al., 2014) was used to infect plants, and the empty vector pKn was used as a control. All clones were transferred into *A. tumefaciens* C58C1 strain for agroinfiltration. Fully expanded *N. benthamiana* leaves (leaves 7 and 8) were co-infiltrated with four different construct combinations: pBin-HMG with pKBG, pBin-HMG with pKn, pKBG with pBin-mCherry, or pKn with pBin-mCherry. Infiltrated leaves (leaves 7 and 8) and systemic leaves (leaves 11 and 12) were collected at 4, 5, and 6 dpi for further analysis. The results were tested in three biological replicates, each of which consisted of three plants.

### RNA Analyses

#### Total RNA Extraction

Total RNA was extracted from leaves using TRIzol (Invitrogen, Carlsbad, CA, United States), purified according to the phenol-chloroform method, and then precipitated in 0.1 volume of 3 M NaOAc and 2.5 volume of 100% ethanol.

#### Northern Blot Analysis

BaMV RNA was detected as described previously (Alazem et al., 2014, 2017). In brief, total RNA (2 μg) was glyoxylated and then separated by electrophoresis on a 1% agarose gel. The RNA was then transferred onto a Hybond-N^+^ membrane (GE Healthcare, Little Chalfont, Buckinghamshire, United Kingdom), cross-linked under UV light, and hybridized against P^32^-labeled (–)CP to detect (+)BaMV (Lin et al., 1993).

#### Real-Time Quantitative PCR (RTqPCR)

Each RNA sample (2 μg) was treated with RQ1-DNase (Promega, Madison, WI, United States) for 30 min at 37°C. RNA samples were then subjected to first-strand cDNA synthesis with Superscript III (Invitrogen). cDNA was then diluted to a final concentration of 20 ng/μl. Primers used for RTqPCR are listed in Supplementary Table S1. All RTqPCR reactions were performed with SYBR Green Supermix (Applied Biosystems, Foster City, CA, United States), following the manufacturer’s instructions. RTqPCR was carried out on three biological replicates, with three technical replicates for each biological replicate.

### Protein Blot

Total protein from *N. benthamiana* leaves was extracted as described previously (Alazem et al., 2014). Briefly, leaves (approximately 0.1 g) were ground to fine powder in liquid nitrogen and homogenized by adding a similar volume of extraction buffer [0.1 M glycine NaOH (pH 9.0), 0.1 M NaCl, 0.5 mM EDTA, 2% sodium dodecyl sulfate (SDS), and 1% sodium laurosarcosine] (Varallyay et al., 2017). Samples were boiled for 5 min and subsequently centrifuged at 13,000 rpm for 5 min. Supernatants were then transferred to new tubes. For BaMV CP detection, crude protein extract (25 μl) was loaded with 2 × dye (1 M Tris, 10% SDS, 100% glycerol, and 900 ml of β-mercaptoethanol in 50 ml of H_2_O) onto 10% SDS-polyacrylamide gels for Western blot analysis, and membranes were hybridized with BaMV anti-CP sera (Lin et al., 1992), anti-HA, anti-Histone H2, anti-Tubulin, or anti-actin (Sigma).

Nuclear fractionation was performed based on the protocol described by Kinkema et al. (2000). Briefly, *N. benthamiana* leaves were homogenized in Honda buffer (2.5% Ficoll 400, 5% dextran T40, 0.4 M sucrose, 25 mM Tris-HCl, pH 7.4, 10 mM MgCl_2_, 10 mM β-mercaptoethanol, and a proteinase inhibitor cocktail) by using a mortar and pestle and then filtered through 62-μm (pore-size) nylon mesh. Triton X-100 was added to a final concentration of 0.5%, and the mixture was incubated on ice for 15 min. The solution was centrifuged at 1,500 g for 5 min, and the pellet was washed with Honda buffer containing 0.1% Triton X-100. The pellet was resuspended gently in 1 ml of Honda buffer and transferred to a microcentrifuge tube. This nucleus-enriched preparation was centrifuged at 100 g for 1 min to pellet starch and cell debris. The supernatant was centrifuged subsequently at 1,800 g for 5 min to pellet the nuclei (Kinkema et al., 2000).

### Yeast Two-Hybrid Assay

Yeast two-hybrid assays were performed according to protocols of the manufacturers of the GAL4 Two-Hybrid Phagemid Vector kits (Agilent Technologies, Inc., 2011). The full-length coding sequences of NbHMG1/2a, BaMV-CP and BaMV-TGBp1 were cloned downstream of the GAL4 activation domain (AD) or GAL4 DNA-binding domain (BD). Rich medium yeast extract, peptone, dextrose (YPD) was used to grow yeast under non-selective conditions. To test the interaction between NbHMG1/2a and CP or TGBp1, we coexpressed the constructs in YRG-2 yeast cells, and selected by incubation in leucine-tryptophan-histidine medium at 28°C for 2–3 days until colonies appeared. Each experiment was repeated three times.

### Confocal Microscopy

To view the subcellular localization of NbHMG1/2a, *N. benthamiana* leaves were infiltrated with Agrobacterium strain C58C1 carrying pBin-mCherry or pBin-mCherry-HMG, and images were taken by confocal microscopy at various timeframes after infiltration. In some agroinfiltrated leaves, pBin-eGFP-Fib was co-expressed as a nucleolar marker. For BaMV infection, leaves were first agroinfiltrated with pKBG (OD, 0.005), agroinfiltrated with pBin-mCherry or pBin-mCherry-HMG (OD, 0.5) 5 days later, and leaves were then observed after a further 2 days. To visualize nuclei in leaf epidermal cells, DAPI (4′,6-diamidino-2-phenylindole) was infiltrated into *N. benthamiana* leaves and then leaves were visualized immediately using a Zeiss LSM510 laser scanning microscope with a 403/1.2 W Korr UV-VIS-IR objective lens. Images were captured using LSM510 software with filters for DAPI (excitation/emission: 405 nm/480–510 nm), GFP (excitation/emission: 488 nm/505–575 nm), and mCherry (excitation/emission: 543 nm/560–615 nm). All images were processed and cropped using Zeiss LSM Image Browser and Photoshop CS5 (Adobe).

## Data Availability Statement

The original contributions presented in the study are included in the article/Supplementary Material, further inquiries can be directed to the corresponding author/s.

## Author Contributions

MA and N-SL designed the research. MA, M-HH, C-HC, and NC performed the research. MA and N-SL analyzed the data and wrote the manuscript which was approved by all authors.

## Conflict of Interest

The authors declare that the research was conducted in the absence of any commercial or financial relationships that could be construed as a potential conflict of interest.

## Footnotes

1 http://nls-mapper.iab.keio.ac.jp

2 http://www.compbio.dundee.ac.uk/www-nod/

3 https://www.ncbi.nlm.nih.gov/Structure/cdd/wrpsb.cgi

## Supplementary Material

Supplementary Figure 1 Sequence similarity between NbHMG1/2 a and b for the coding region (CDS), 5′ UTR, and 3′ UTR.

Supplementary Figure 2 NbHMG1/2a localization in BaMV-infected *N. benthamiana* at 1.5 dpi. *N. benthamiana* leaves were co-agroinfiltrated with pKBG or empty vector (pKn) combined with either pBin-HA-mCherry or pBin-HA-mCherry-HMG, as described in the legend of Figure 6. Confocal microscopic observation was carried out at 1.5 dpi. Scale bars represent 50 μm. The experiment was repeated three times with similar results, and representative images are shown.

Supplementary Figure 3 NbHMG1/2a localization in BaMV-infected *N. benthamiana* at 2 dpi. The experiment was carried out as described in Supplementary Figure S2, except confocal analyses were conducted at 2 dpi.

Supplementary Figure 4 NbHMG1/2a localization in BaMV-infected *N. benthamiana* at 3 dpi. The experiment was carried out as described in Supplementary Figure S2, except confocal analyses were conducted at 3 dpi.

Supplementary Figure 5 Expression levels of *BaMV-RDR*, *TRV-RDR*, and *NbHMG1/2a in N. benthamiana* plants silenced with *NbHMG1/2a*. Transcript levels of *BaMV-RDR* **(A)**, *TRV-RDR* **(B)**, and *NbHMG1/2a* **(C)** in BaMV-inoculated *N. benthamiana* leaves at 6 dpi. Plants were already infiltrated with the empty TRV-silencing vector (EV), or with TRV-HMG (HMGi) 7–8 days before BaMV infection. Statistical analysis was carried out as described in Figure 3, with ^∗^ and ^∗∗^ representing *P* < 0.05 and < 0.01, respectively.

Supplementary Figure 6 Expression of BaMV-RDR in *N. benthamiana* plants transiently expressing NbHMG1/2a-mCherry. Transcript levels of *BaMV-RDR* in BaMV-inoculated *N. benthamiana* leaves at 6 dpi in control plants (pBin-mCherry) and NbHMG1/2a-mCherry expressing plants (pBin-HMG). Statistical analysis was carried out as described in Figure 3, with ^∗∗^ representing *P* < 0.01.

Supplementary Figure 7 Protein blot for HA-mCherry (30 kDa) or HA-HMG-mCherry (45 kDa) in leaves infiltrated with the control vector (pKn). Leaves from *N. benthamiana* plants were collected at 4, 5, and 6 dpi for analysis. The experiment was carried out as indicated in the legend of Figure 5, and was repeated three times with similar results. Ponceau S was used as a loading control.

Supplementary Figure 8 NbHMG1/2a localization in BaMV-infected *N. benthamiana*. Leaves of *N. benthamiana* were first agroinfiltrated with pKBG (BaMV) or pKn (vector), and then agroinfiltrated with mCherry or mCherry-HMG 5 days later. Samples were collected 2 days after mCherry or mCherry-HMG expression for confocal microscopic observation. DAPI was infiltrated into *N. benthamiana* leaves before observation to detect nuclei. Scale bars represent 20 μm. The experiment was repeated three times with similar results, and representative images are shown.

Supplementary Figure 9 Yeast two-hybrid assay of HMG against BaMV proteins. Binding assays were performed for HMG against BaMV CP and TGBp1 proteins. Yeast cells were grown in liquid media to an OD_600_ of 0.1 and then subjected to a 10^–1^, 10^–2^, and 10^–3^ dilution series. Ten microliters of liquid medium were grown on SD medium lacking Trp and Leu, or lacking Trp, Leu, and His. Images were taken 3 days after incubation.

Supplementary Table 1 Primers used in the study.

Supplementary Table 2 Probable targets of silencing if the first 300 bp of HMG1/2a is targeted for silencing.
