# Supplementary figures and images for: Disrupting the Homeostasis of High Mobility Group Protein Promotes the Systemic Movement of Bamboo mosaic virus
Source: Front Plant Sci. 2020 Dec 16;11:597665. doi: 10.3389/fpls.2020.597665 (PMC7793662; doi:10.3389/fpls.2020.597665)

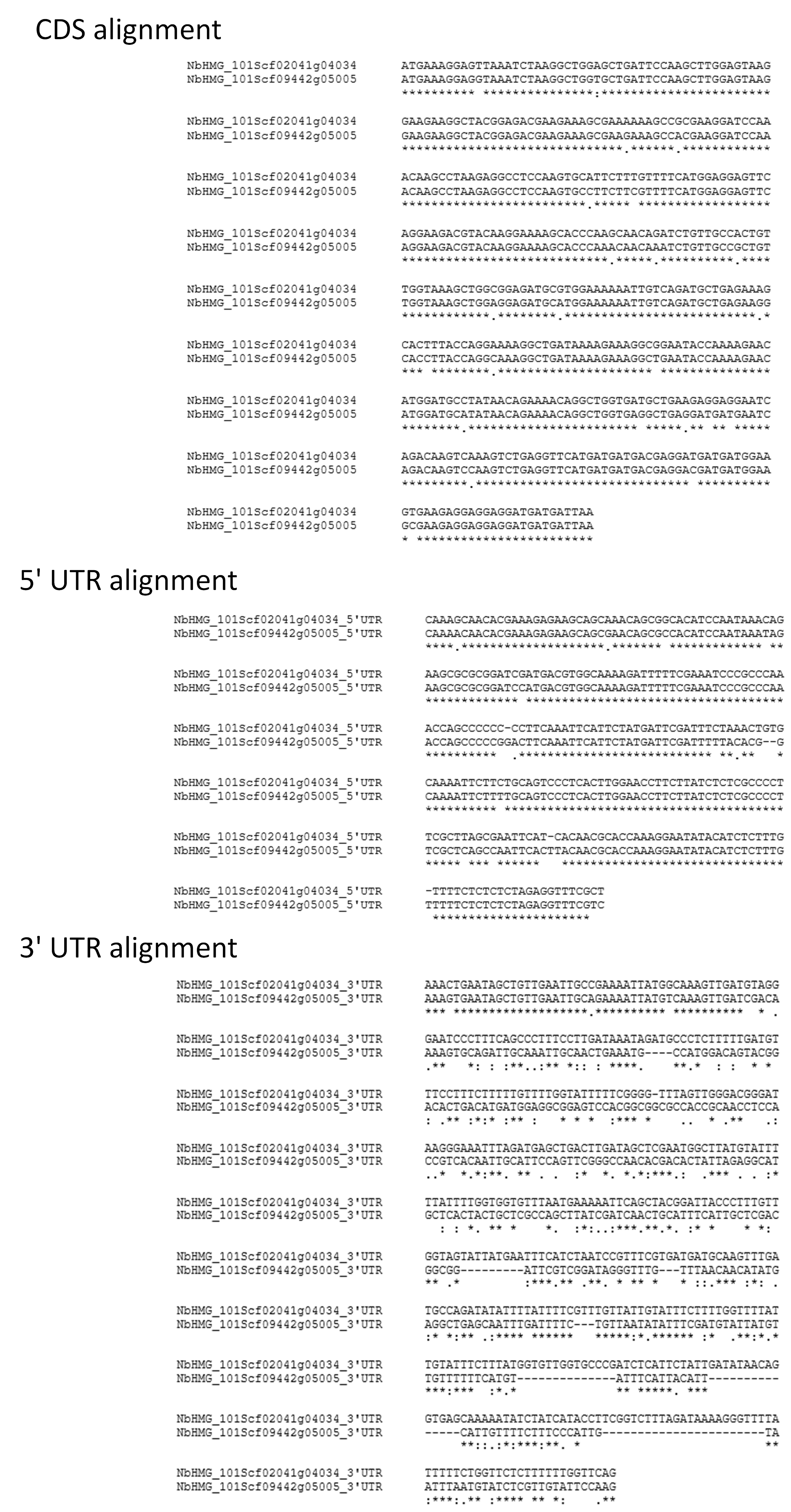

Supplement: Supplementary Figure 1 — Sequence similarity between NbHMG1/2 a and b for the coding region (CDS), 5′ UTR, and 3′ UTR. [file Image_1.JPEG]

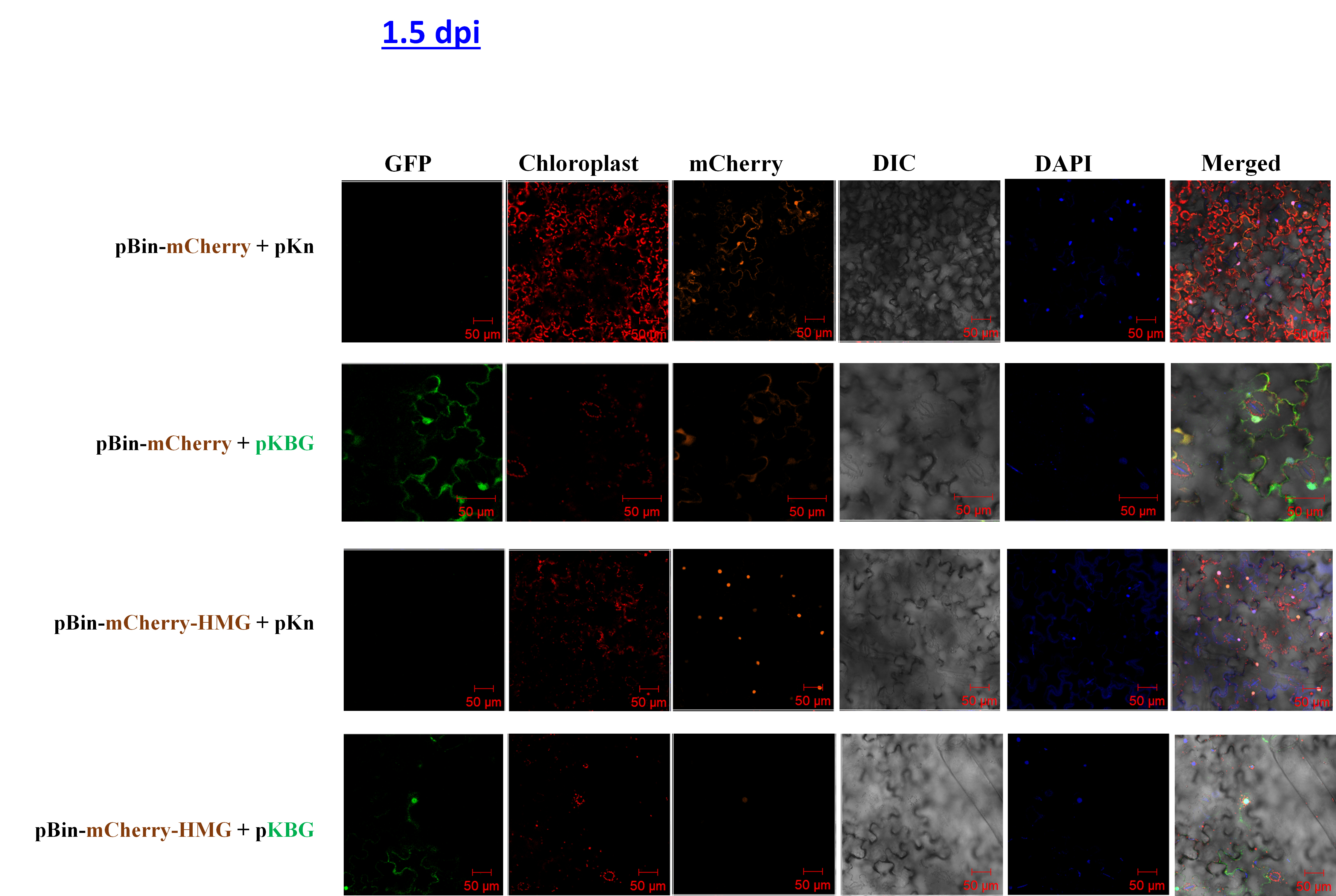

Supplement: Supplementary Figure 2 — NbHMG1/2a localization in BaMV-infected N. benthamiana at 1.5 dpi. N. benthamiana leaves were co-agroinfiltrated with pKBG or empty vector (pKn) combined with either pBin-HA-mCherry or pBin-HA-mCherry-HMG, as described in the legend of Figure 6. Confocal microscopic observation was carried out at 1.5 dpi. Scale bars represent 50 μm. The experiment was repeated three times with similar results, and representative images are shown. [file Image_2.JPEG]

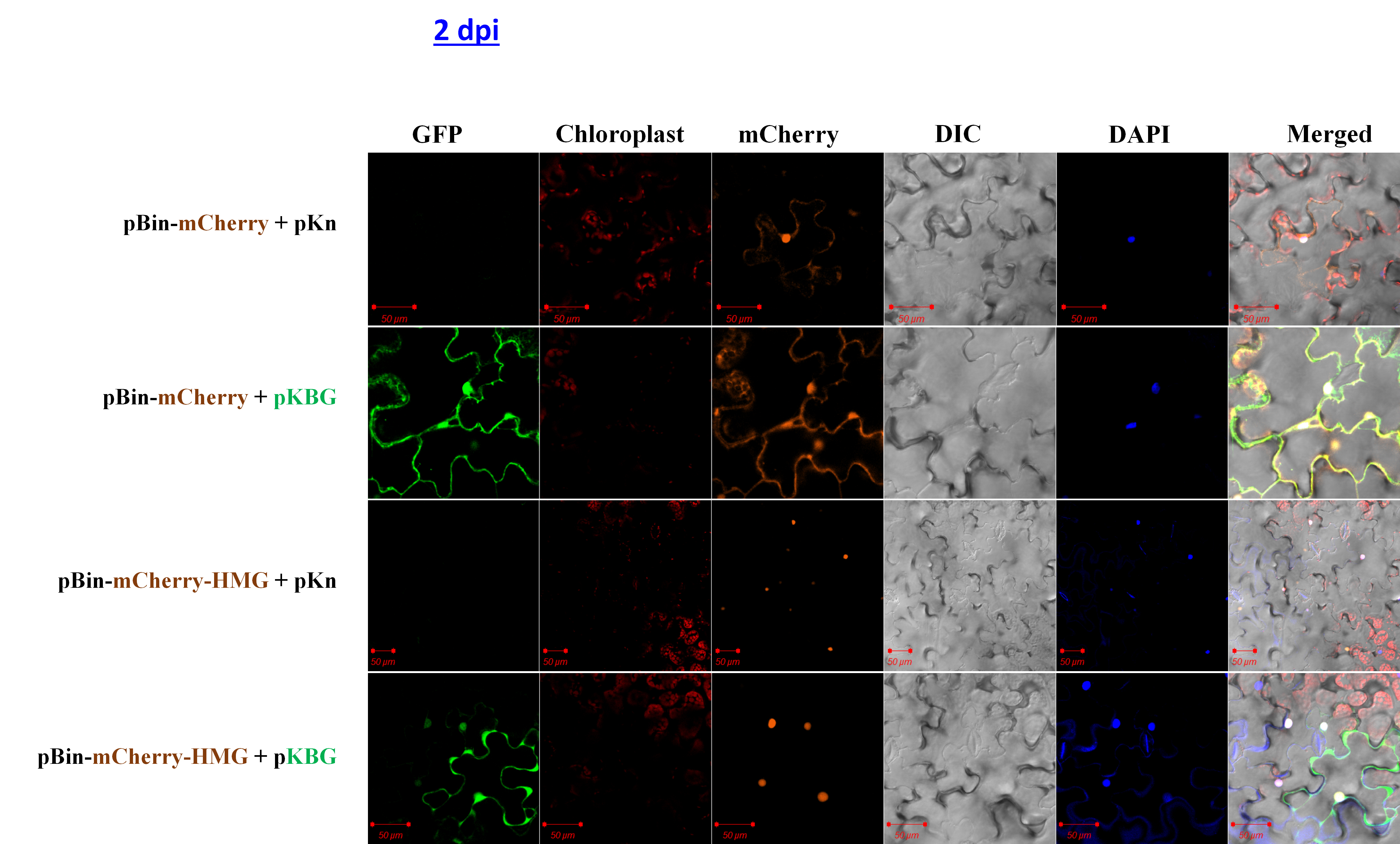

Supplement: Supplementary Figure 3 — NbHMG1/2a localization in BaMV-infected N. benthamiana at 2 dpi. The experiment was carried out as described in Supplementary Figure S2, except confocal analyses were conducted at 2 dpi. [file Image_3.JPEG]

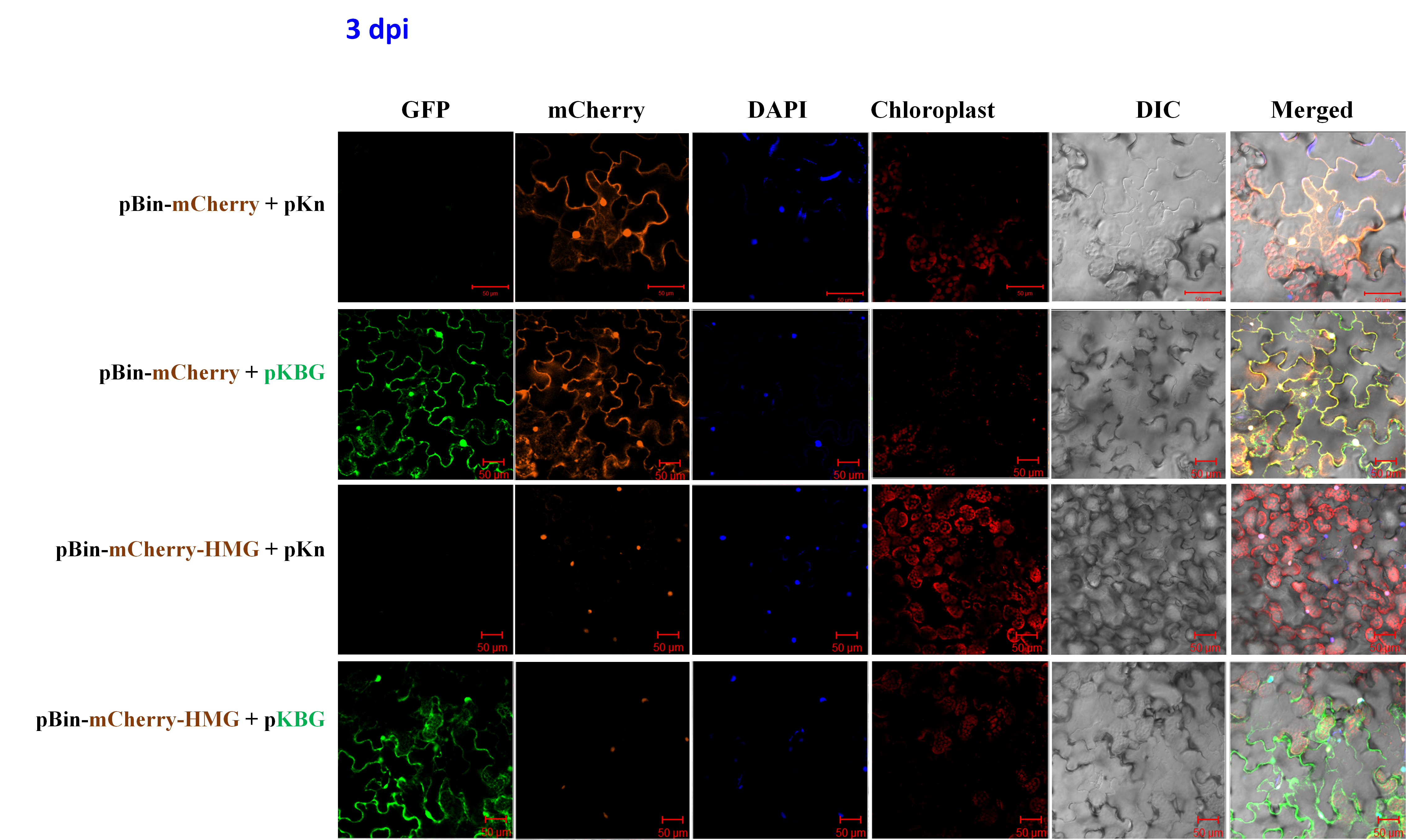

Supplement: Supplementary Figure 4 — NbHMG1/2a localization in BaMV-infected N. benthamiana at 3 dpi. The experiment was carried out as described in Supplementary Figure S2, except confocal analyses were conducted at 3 dpi. [file Image_4.JPEG]

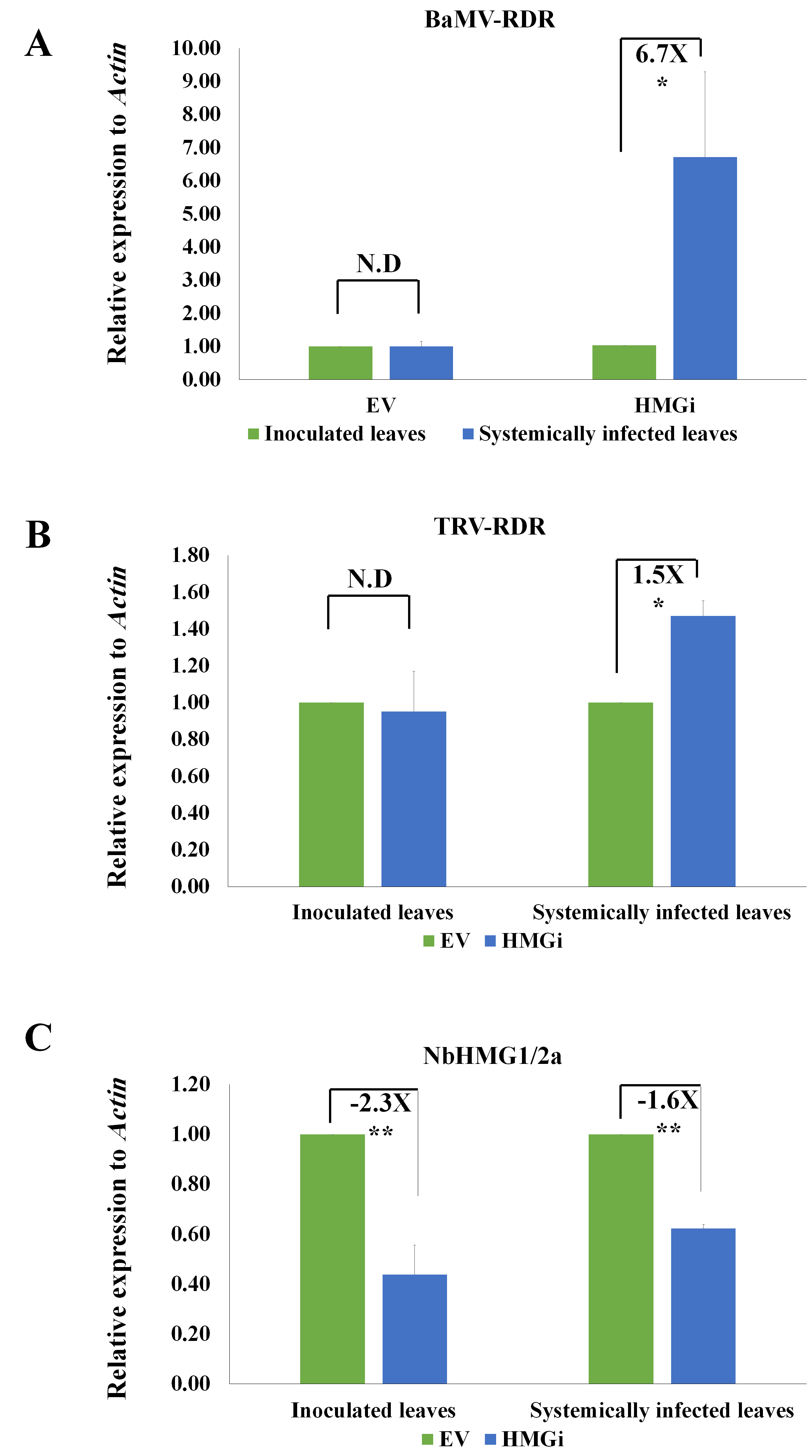

Supplement: Supplementary Figure 5 — Expression levels of BaMV-RDR, TRV-RDR, and NbHMG1/2a in N. benthamiana plants silenced with NbHMG1/2a. Transcript levels of BaMV-RDR (A), TRV-RDR (B), and NbHMG1/2a (C) in BaMV-inoculated N. benthamiana leaves at 6 dpi. Plants were already infiltrated with the empty TRV-silencing vector (EV), or with TRV-HMG (HMGi) 7–8 days before BaMV infection. Statistical analysis was carried out as described in Figure 3, with ∗ and ∗∗ representing P < 0.05 and < 0.01, respectively. [file Image_5.JPEG]

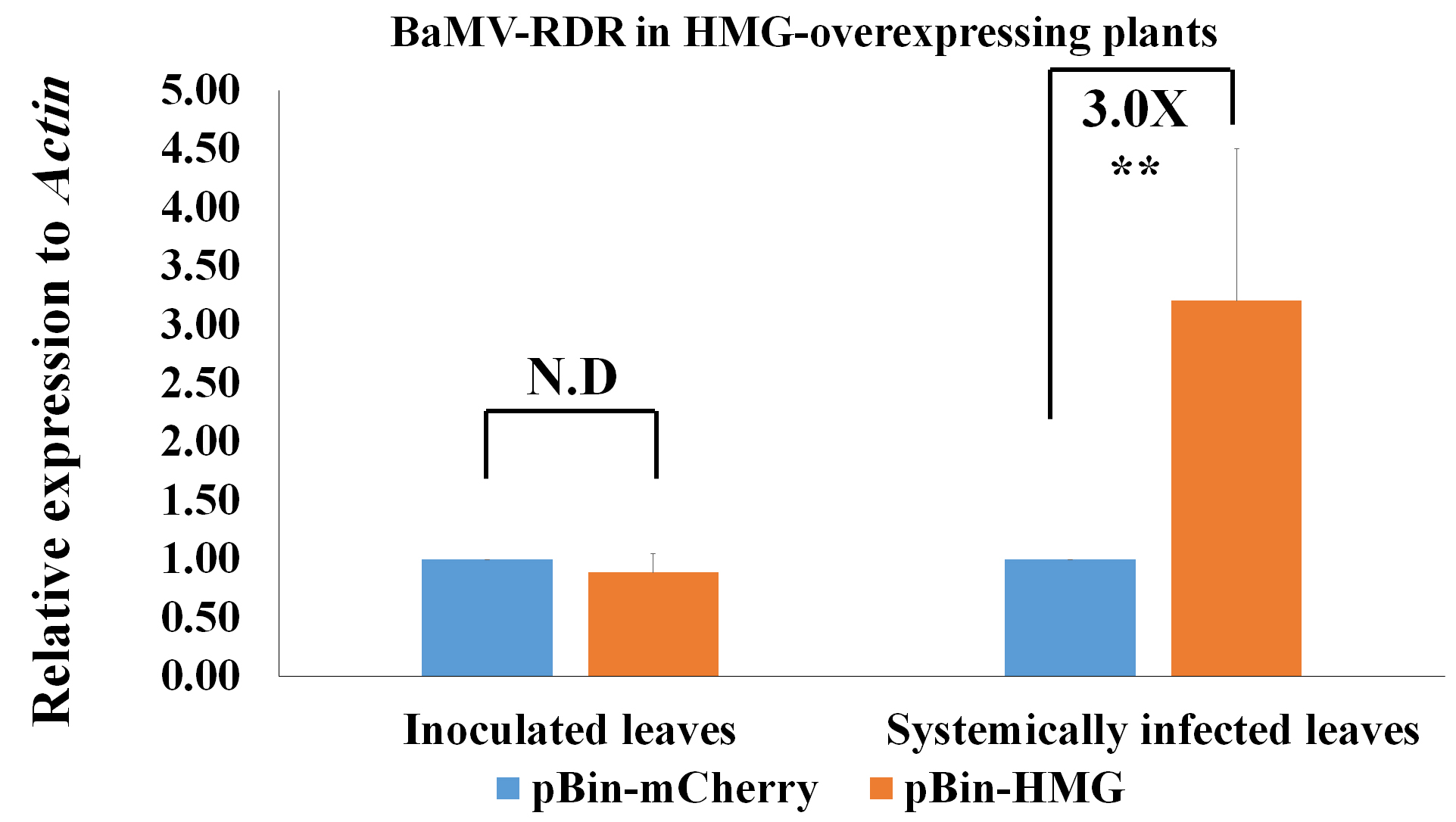

Supplement: Supplementary Figure 6 — Expression of BaMV-RDR in N. benthamiana plants transiently expressing NbHMG1/2a-mCherry. Transcript levels of BaMV-RDR in BaMV-inoculated N. benthamiana leaves at 6 dpi in control plants (pBin-mCherry) and NbHMG1/2a-mCherry expressing plants (pBin-HMG). Statistical analysis was carried out as described in Figure 3, with ∗∗ representing P < 0.01. [file Image_6.JPEG]

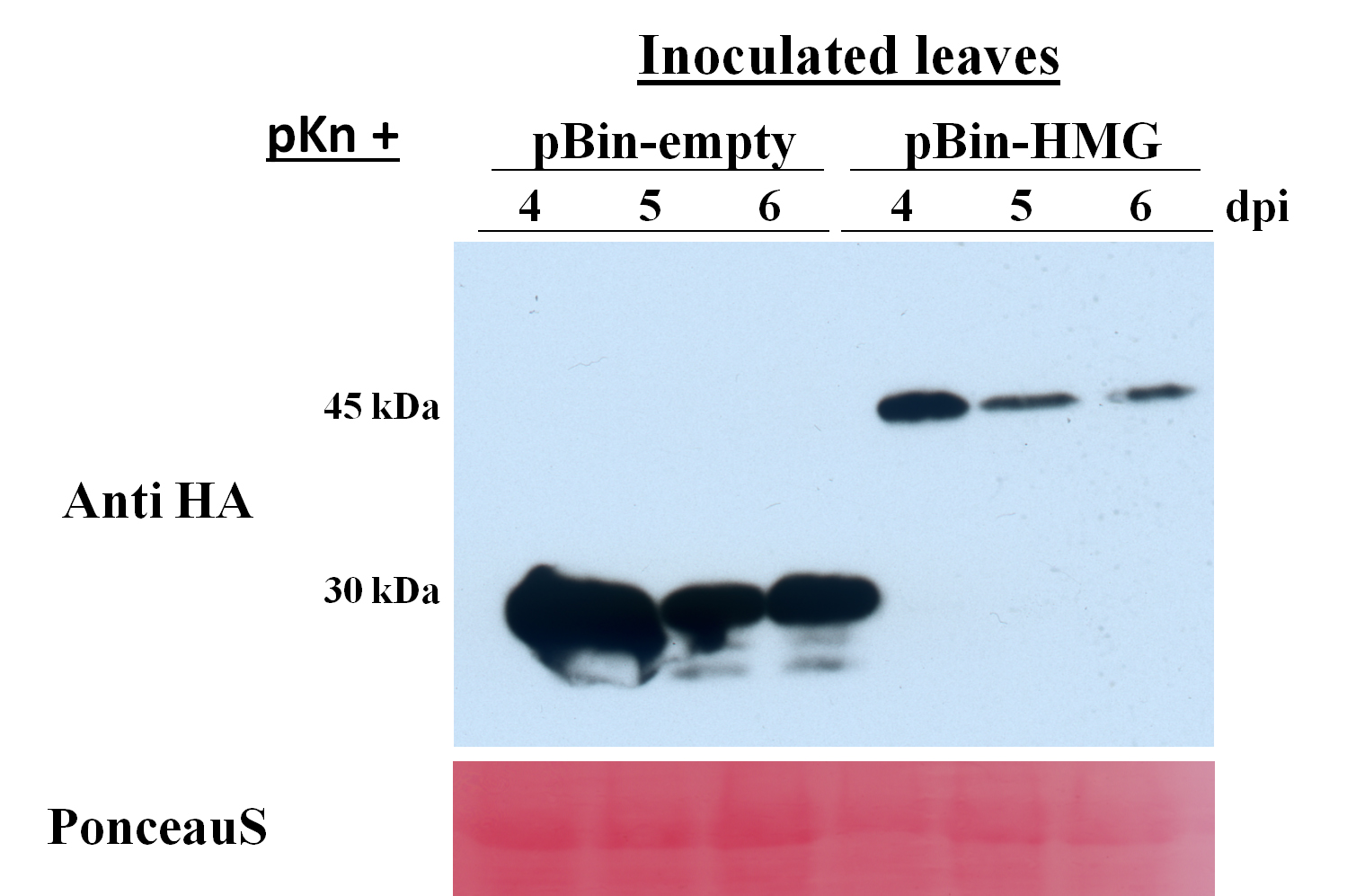

Supplement: Supplementary Figure 7 — Protein blot for HA-mCherry (30 kDa) or HA-HMG-mCherry (45 kDa) in leaves infiltrated with the control vector (pKn). Leaves from N. benthamiana plants were collected at 4, 5, and 6 dpi for analysis. The experiment was carried out as indicated in the legend of Figure 5, and was repeated three times with similar results. Ponceau S was used as a loading control. [file Image_7.JPEG]

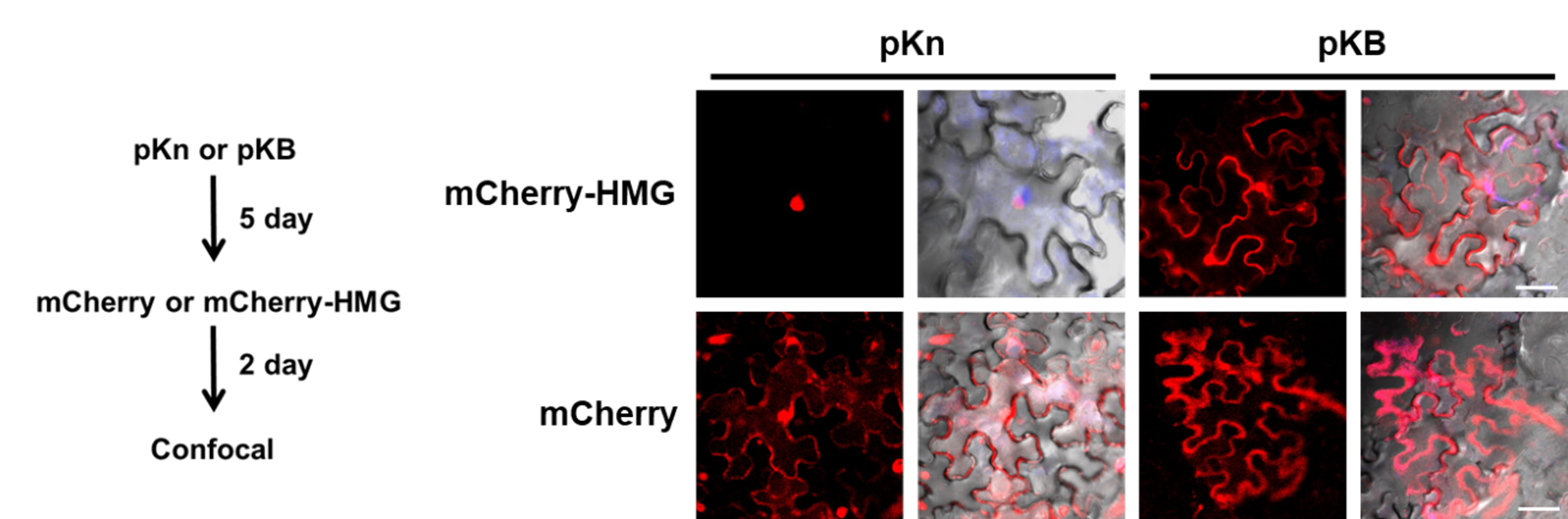

Supplement: Supplementary Figure 8 — NbHMG1/2a localization in BaMV-infected N. benthamiana. Leaves of N. benthamiana were first agroinfiltrated with pKBG (BaMV) or pKn (vector), and then agroinfiltrated with mCherry or mCherry-HMG 5 days later. Samples were collected 2 days after mCherry or mCherry-HMG expression for confocal microscopic observation. DAPI was infiltrated into N. benthamiana leaves before observation to detect nuclei. Scale bars represent 20 μm. The experiment was repeated three times with similar results, and representative images are shown. [file Image_8.JPEG]

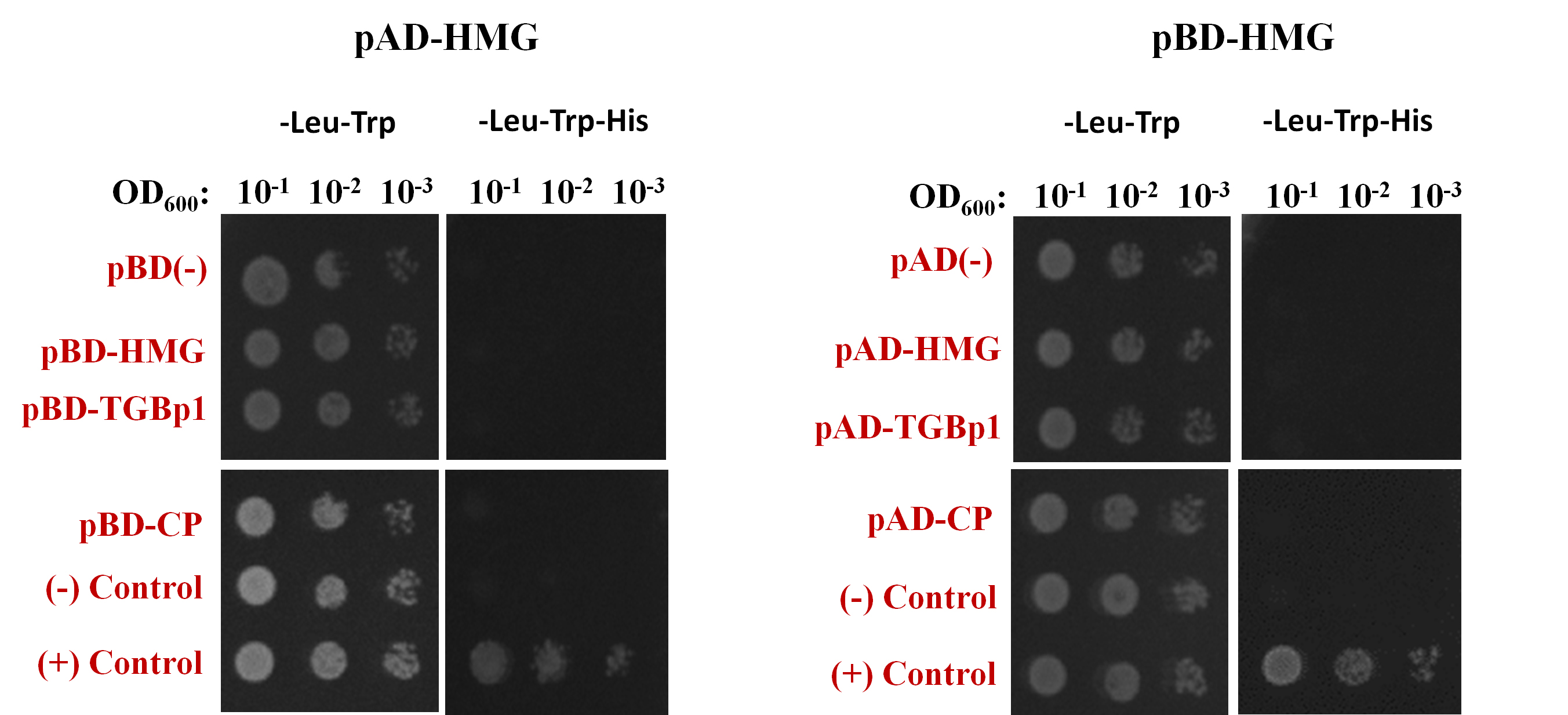

Supplement: Supplementary Figure 9 — Yeast two-hybrid assay of HMG against BaMV proteins. Binding assays were performed for HMG against BaMV CP and TGBp1 proteins. Yeast cells were grown in liquid media to an OD600 of 0.1 and then subjected to a 10–1, 10–2, and 10–3 dilution series. Ten microliters of liquid medium were grown on SD medium lacking Trp and Leu, or lacking Trp, Leu, and His. Images were taken 3 days after incubation. [file Image_9.JPEG]
